# Autophagy-Inflammation Interplay During Infection: Balancing Pathogen Clearance and Host Inflammation

**DOI:** 10.3389/fphar.2022.832750

**Published:** 2022-02-22

**Authors:** Yuqian Pang, Lanxi Wu, Cheng Tang, Hongna Wang, Yongjie Wei

**Affiliations:** ^1^ Affiliated Cancer Hospital and Institute of Guangzhou Medical University, Guangzhou, China; ^2^ Key Laboratory for Cell Homeostasis and Cancer Research of Guangdong Higher Education Institutes, Guangzhou, China; ^3^ GMU-GIBH Joint School of Life Sciences, Guangzhou Medical University, Guangzhou, China; ^4^ State Key Laboratory of Respiratory Disease, National Clinical Research Center for Respiratory Disease, Guangzhou Institute of Respiratory Health, Guangzhou, China

**Keywords:** autophagy, virus, infection, bacteria, microbial, inflammation, inflammasome

## Abstract

Inflammation is an essential immune response of the host against infections but is often over-activated, leading to a variety of disorders. Autophagy, a conserved degradation pathway, also protects cells by capturing intracellular pathogens that enter the cell and transporting them to the lysosome for clearance. Dysfunctional autophagy is often associated with uncontrolled inflammatory responses during infection. In recent years, more and more research has focused on the crosstalk between autophagy and inflammation. In this paper, we review the latest research advances in this field, hoping to gain insight into the mechanisms by which the body balances autophagy and inflammation in infections and how this mechanism can be used to fight infections better.

## Introduction

Infectious diseases are caused by pathogenic microorganisms such as bacteria, viruses and parasites. From the moment a pathogen invades, it triggers a series of battles between the host’s defense system and itself (Mitchell and Isberg, 2017). One of these battles is inflammation, an innate immune response initiated by the recognition and interaction of host pattern recognition receptors (PRRs) with pathogenic microbial components such as bacterial lipopolysaccharide (LPS), flagellin, and viral DNA and RNA. Upon initiation of the inflammatory signaling, transcription factors such as interferon regulatory factors (IRFs) and nuclear factor-kappa B (NF-κB) are transported to the nucleus to drive the synthesis of two sets of anti-pathogen products. One group of them, the antimicrobial peptides and complement components, directly attack and destroy the pathogens. The other set of products, including pro-inflammatory cytokines such as interleukin (IL) and tumor necrosis factors (TNFs), fight pathogens by activating endothelial cells and recruiting innate and adaptive immune cells to the site of infection (Smale, 2010).

In addition to inflammation, studies have shown that autophagy is also involved in the defense against pathogen invasion (Mao and Klionsky, 2017; Choi et al., 2018). Autophagy is an evolutionarily conserved process by which cellular material is encapsulated within an isolated membrane structure called autophagosome and transported to lysosomes for degradation. Autophagy can indiscriminately engulf cytoplasmic components, referred to as bulk autophagy. It can also selectively break down redundant or dysfunctional cellular components, such as misfolded or aggregated proteins, damaged organelles, and pathogens that have invaded the cell. Selective autophagy that targets intracellular pathogens for degradation has been given the proprietary name xenophagy and is the most direct example of autophagy against pathogenic invasion. Xenophagy and other forms of selective autophagy that eliminate unnecessary cellular components are similarly processed (Jin et al., 2013; Xu et al., 2019). They are both internal quality control mechanisms of the cell and play an essential role in maintaining cellular homeostasis.

Given the critical role of autophagy in pathogen clearance, its dysfunction often leads to excessive host inflammation and damage during infection (Deretic, 2021). Both autophagy and inflammation can be activated by pathogens, and their crosstalk occurs continuously as the host fights infection. A growing number of studies have suggested that autophagy can fine-tune the inflammatory responses induced by pathogens or their harmful components (Levine et al., 2011). These fine-tunings are manifested in several modalities: Firstly, the removal of pathogens by autophagy eliminates the source of provocation and thus suppresses inflammation, whereas hijacking of autophagy by pathogens leads to more severe inflammation. Secondly, autophagy may directly regulate inflammation by removing or down-regulating pro-inflammatory cytokines and degrading inflammasome components (e.g., absent in melanoma 2 (AIM2)). And lastly, autophagy can regulate the function of organelles within immune cells, thereby indirectly influencing inflammatory factor production (Deretic, 2021). Although most past studies have focused on the role of autophagy in the regulation of inflammation, a growing body of work suggests that multiple components of inflammatory signaling during infection also regulate different steps of autophagy. A better understanding of the autophagy/inflammation cascade would be beneficial in the fight against infectious diseases. However, many questions remain as to how the host controls the activation and balance of autophagy and inflammation through multiple mechanisms in various infection settings (Matsuzawa-Ishimoto et al., 2018). With this in mind, this paper will review recent advances on how autophagy and inflammation crosstalk during bacterial and viral invasion and then discuss how the balance of their interaction will affect host cell homeostasis and how it can be leveraged to fight infection better.

## Pathogenic Infection and Inflammation

Microorganisms are present in every corner of the earth, including inside the human body, which brings us into frequent contact with them. Therefore, the host inflammatory response to a microbial invasion often occurs at high frequency (Nathan and Ding, 2010). Inflammation is a protective mechanism of the body activated by microbial infection or tissue damage (Medzhitov, 2010). It is triggered mainly by recognition of the conserved structures of pathogen-associated molecular patterns (PAMPs) of invading pathogens or damage-associated molecular patterns (DAMPs) of endogenous substances by PRRs of innate immune cells, such as macrophages, fibroblasts, mast cells, dendritic cells, circulating leukocytes (including granulocytes and neutrophils), etc. PAMPs are derived from microorganisms and thus drive inflammation in response to infection. They are non-specific but structurally conserved molecules common on the surface of microbes and their derivatives but not in host cells. Some common PAMPs are LPS and peptidoglycan (PGN) on the surface of Gram-negative bacteria, DNA and RNA of viruses. DAMPs are derived from host cells, including tumor cells, dead or dying cells, or products released from cells in response to signals such as hypoxia. Because they are derived from host materials, DAMPs induce what’s known as sterile inflammatory responses and thus are not the focus of this review.

When inflammatory signaling is initiated, immune cells are activated at the site of infection to remove invading pathogens and some host debris through phagocytosis. They also secrete pro-inflammatory cytokines such as IL-1, IL-6, IL-12, and TNFs to control the infection. Thus, the outcome of inflammation is theoretically to prevent the spread of infection and subsequent restoration of tissue function (Medzhitov, 2008). However, during pathogen invasion, inflammation is often over-activated, leading to damage to the host. Influenza viral infection of both the Spanish strain of 1918 and the H5N1 strain recruited excess inflammatory leukocytes to the lungs, leading to excessive cytokine secretion and a dramatic increase in infection and mortality rates (Iwasaki and Medzhitov, 2011). SARS-CoV-1 ORF3a, ORF8b and E proteins have been reported to enhance activation of the inflammasome, leading to increased secretion of IL-1 and IL-18, and subsequent pathological changes associated with inflammation. Similarly, the NSP9 and NSP10 proteins of SARS-CoV-2 induce overproduction of IL-6 and IL-8, which are the leading causes of cytokine storm leading to death in COVID-19 patients (Ramanathan et al., 2020). It is thus clear that excessive activation of inflammation harms rather than protects the organism and therefore needs to be finely regulated. For example, lipid mediators derived from long chain polyunsaturated fatty acids (LC-PUFAs) such as arachidonic acid (AA) and omega-3 polyunsaturated fatty acids play an important role in the regulation of inflammation. Specialized pro-resolving lipid mediators (SPMs), including lipoxin (LX), resolvin (RvE), protectin and maresins, can limit pro-inflammatory cytokines production and promote inflammation regression (Serhan, 2014; Serhan and Levy, 2018).

Inflammation is a complex process involving many aspects, including the composition of immune cells in tissues, the sequential expression of inflammation-related genes, and the transduction of inflammatory signals. Thus, inflammation can be regulated accordingly at three temporally and spatially inextricably linked levels: cell-specific, transcription-specific and signaling-specific (Medzhitov and Horng, 2009). An example of cell-specific regulation is that macrophages are the primary effector cells of inflammation; they phagocytose and destroy invading pathogens and associated substances that damage the host and mediate the inflammatory response by secreting cytokines and chemokines (Galli and Saleh, 2021). In a standard model for studying inflammatory regulation, hundreds of genes are repressed or induced in macrophages upon stimulation of their specific PRR Toll-like receptor 4 (TLR4) by LPS. This complex transcriptional response is an example of the transcription-specific regulation of inflammation (Medzhitov and Horng, 2009). Similarly, the inflammasome, a signaling platform for pro-inflammatory factor activation, is also subject to transcription-specific regulation (Christgen et al., 2020). In terms of signal-specific inflammatory regulation, NF-κB-mediated cytokine production is one of the most common signal pathways in infection. These three levels of inflammation-specific regulation are intertwined without strict boundaries, and it is not easy to distinguish them in most cases (Lawrence, 2009; Liu et al., 2017a).

## Pathogenic Infection and Autophagy

### Overview of Autophagy

**FIGURE 1:**
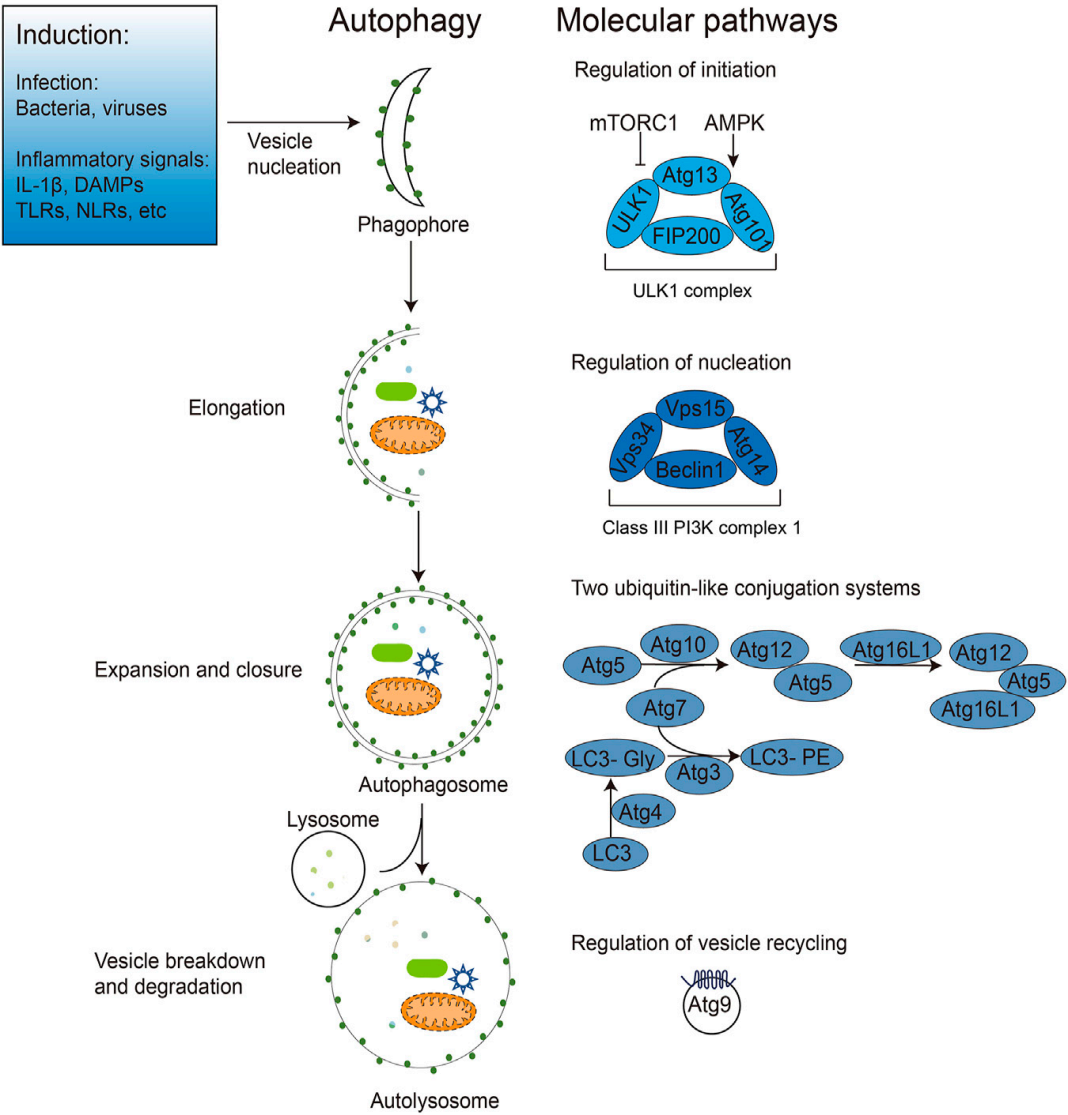
Induction and regulation of autophagy during pathogen infection. Bacterial, viral, and inflammatory signals can all induce autophagy initiation. After autophagy initiation, dispersed intracellular membrane vesicles are induced to nucleate and form phagophores. The nucleation induction is regulated by mTOR, AMPK and ULK1 (Atg1 in yeast) and the Class III PI3K complexes containing Beclin1 (Atg6 in yeast), Atg14 and other proteins. The phagophore extends and wraps around the autophagic cargos (e.g., intracellular bacteria and viruses, DAMPs), expanding and closing under two ubiquitin-like Atg5-Atg12 and Atg8 (LC3 in mammals) ligation systems to form the bilayer membrane structure of autophagosomes. Finally, autophagosomes fuse with lysosomes to form the monolayer autophagolysosomes, degrading the enclosed cargos and recycling membrane structures with Atg9.

Autophagy is a highly conserved cellular degradation process that sequesters a portion of the cytoplasm and organelles in double-membrane autophagosomes, which are then transported to lysosomes for breakdown and recycling. Autophagy includes macroautophagy, microautophagy and chaperone-mediated autophagy, of which macroautophagy is often referred to as autophagy (Mizushima and Komatsu, 2011). The dynamic process of autophagy can be broken down into several steps: initiation, cargo recognition and packaging, vesicle nucleation, vesicle expansion and closure, vesicle/lysosome fusion, vesicle breakdown and recycling of the autophagy-related molecules (Figure 1). With the rapid development of genetic techniques, the molecular mechanisms and related genes involved in autophagy were revealed first in yeast and then in mammals. To date, 41 autophagy genes have been identified in yeast, and nearly half of them are well conserved across multicellular species such as Drosophila, nematodes, and mammals. The proteins encoded by these genes are uniformly referred to as Autophagy-related (ATG) proteins, 20 of which constitute the core autophagy machinery. According to the specific steps of their involvement in the autophagic process, ATG proteins can be divided into the following groups: Protein kinase complexes Atg1 (mammalian ULK1 homologue), Atg13, and Atg17, which act at the onset of autophagy; Lipid kinase complexes Atg6 (mammalian Beclin one homologue), Atg14, Atg34, and Vps15, which mediate phagosome formation; Ubiquitin-like ligation systems, including Atg3, Atg7, Atg5, Atg12, and Atg8 (mammalian microtubule-associated protein one light chain 3 (LC3) homologue), which promote phagosome expansion and autophagosome maturation; Tethering complexes Atg2, Atg9, and Atg18, which detach and recycle Atg proteins from autophagosomes after autophagosome maturation; and the vacuolar integral membrane protein Atg22, which promotes amino acid efflux from degraded autophagosomes (Levine and Kroemer, 2008; Kroemer, 2019).

Based on the selectivity of their cargo, autophagy can be divided into non-selective and selective autophagy. Non-selective autophagy, also known as bulk autophagy, is an emergency response mechanism of cells to starvation or stress. It randomly engulfs and digests substances in the cytoplasm for rapid recycling to replenish the nutrient deficit absorbed from the environment to maintain the cell’s most basic survival needs. Selective autophagy is a cellular self-quality control mechanism that selectively degrades protein aggregates, damaged organelles, excess peroxisomes, and invading pathogens to maintain homeostasis within a nutrient-rich cell. Autophagy achieves its selectivity mainly through the specific binding of selective autophagy receptors (SLRs), such as SQSTM1/p62, NBR1, OPTN, NDP52 and TAX1BP1, on the surface of the degraded substrate and LC3 on the autophagosome surface (Green and Levine, 2014). These autophagy receptors all contain one or more [W/F/Y]xx [L/I/V] LIR motifs that bind specifically to LC3 and direct degradation targets to the autophagosome (Li et al., 2021b).

### Autophagy in Direct Pathogen Elimination

A specific type of selective autophagy, termed xenophagy, can directly remove invading pathogens, such as *Mycobacterium tuberculosis* that resides within the phagosome (Gutierrez et al., 2004), *Shigella* that escapes from the phagosome into the cytoplasm (Ogawa et al., 2005), and *group A Streptococci* that invade into the host cell (Nakagawa et al., 2004). Other intracellular bacteria such as *Listeria monocytogenes*, *Salmonella enterica*, *Francisella tularensis* and *Pseudomonas* spp. have also been reported to be directly eliminated by xenophagy (Orvedahl and Levine, 2009). Among the viruses, herpes simplex virus 1 (HSV-1) and Sindbis virus have been well demonstrated to be cleared by virophagy, a xenophagy subtype that specifically targets the virus (Liang et al., 1998; Orvedahl et al., 2010). The SLRs SQSTM1/p62, NDP52 (nuclear dot protein 52 kDa, also known as CALCOCO2) and OPTINEURIN were shown to be responsible for recognizing these pathogens for autophagic degradation (Thurston, 2009; Zheng et al., 2009; Orvedahl et al., 2010; Wild et al., 2011).

In addition to direct attacks on pathogens, autophagy also limits their survival by removing critical replication factors from pathogens. When these replication factors become autophagic cargos, they are often first labeled by ubiquitination and subsequently encapsulated into phagophore via selective autophagic receptors such as p62. Capsid of Chikungunya virus (CHIKV) is targeted by p62 and degraded through autophagy (Judith et al., 2013). Similarly, human immunodeficiency virus 1 (HIV-1) replication in CD4-positive T cells is restricted by p62-mediated selective autophagic degradation of the viral transcriptional activator Tat (Sagnier et al., 2015).

When autophagy clears pathogens and their components, it also inhibits the function of pathogen PAMPs as inflammatory stimuli, thus inhibiting the downstream inflammatory signaling pathway. However, autophagy can also be held hostage by bacteria and viruses, when autophagy will aid pathogen replication and damage the host. *Salmonella*, poliovirus, rhinovirus, coxsackievirus B3 (CVB3), enterovirus 68 (EVD68) and SARS-COV-2 employ autophagy to increase their replication in different context (Huang and Brumell, 2014; Yu et al., 2014; Choi et al., 2018; Miao et al., 2021). Thus autophagy also needs to be finely regulated during pathogen invasion.

### MUTUAL REGULATION OF AUTOPHAGY AND INFLAMMATION

Many inflammatory signaling-related elements such as Toll-like receptors (TLRs), PAMPs, NOD-like receptors (NLRs), pro-inflammatory cytokines, DAMPs and SPMs can activate autophagy (Levine et al., 2011; Ge et al., 2018). TLRs of innate immune cells trigger inflammatory responses by recognizing corresponding PAMPs on endogenous or exogenous ligands. TLR3, TLR4 and TLR7 can induce autophagy after being activated (Delgado et al., 2008), possibly through a mechanism involving competition for Beclin 1 with Trif and MyD88 (TLR adaptor proteins) away from the autophagy inhibitory protein Bcl-2 (Xu et al., 2007; Shi and Kehrl, 2008). TLR4 in macrophages can also directly activate VPS34, which initiates autophagy and targets invading mycobacteria (Xu et al., 2007). In addition, NLRs also induce autophagy. During *Shigella* infection, two important receptor proteins in the NLR family, NOD1 and NOD2, are recruited to the bacterial infection site, and autophagy protein Atg16L1 is recruited simultaneously, triggering autophagy to help remove the bacteria (Travassos et al., 2010). Several studies have reported that IL-1β, an IL-1 family member, also an inflammasome component, induces autophagy in different scenarios (English et al., 2014; Shen et al., 2017; Pan et al., 2020). Examples include 1) IL-1β upregulates autophagy in mouse macrophages after HSV-1 infection (English et al., 2014); 2) IL-1β induces mitochondrial damage in serum-deprived human degenerative myeloid cells, thereby simultaneously triggering mitochondria-dependent apoptosis and cytoprotective autophagy against apoptosis (Shen et al., 2017); 3) Exogenous application of IL-1β induces autophagy in preimplantation embryos through an unknown mechanism (Pan et al., 2020). The induction of autophagy by DAMPs such as reactive oxygen species (ROS), high mobility group box protein 1 (HMGB1) and heat shock protein 60 (HSP60) have also been demonstrated in different studies (Scherz-Shouval et al., 2007; Tang et al., 2012; Liu et al., 2017b). During autophagosome formation, Atg4 must become inactive after the initial cleavage of the Atg8-like protein to ensure the binding of Atg8 to the autophagosome membrane. When cells are starved, the production of ROS, especially H_2_O_2_, is necessary to deactivate Atg4 and promote autophagy (Scherz-Shouval et al., 2007). During ischemia-reperfusion (I/R) injury in the lung, HMGB1 and HSP60 are released as DAMPs of necrotic cells to induce inflammation in alveolar macrophages in an autophagy-dependent manner, although the detailed mechanisms remain to be discovered (Liu et al., 2017a). In addition, some SPMs, such as 15-epi-LXA4 and resolvin D1 (RvD1), had been reported to induce autophagy in mouse and human macrophages (Prieto et al., 2015). Overexpression of cytoplasmic phospholipase A2 (cPLA2), a rate-limiting enzyme that promotes the synthesis of SPMs from AA, induced autophagy in mouse macrophages and primary human peripheral blood mononuclear cells, further validating the function of SPMs in autophagy induction (Qi et al., 2011).

While autophagy is activated by inflammatory components, it also regulates various aspects of inflammation, including activation of inflammatory signals, secretion of pro-inflammatory cytokines, activation or inhibition of inflammasomes, and composition of immune cells in tissues. An excellent example of inflammatory signaling being regulated by autophagy is the NF-κB signaling. When autophagy is defective, p62, the selective autophagic adapter and substrate, accumulates in cells and activates the pro-inflammatory transcription factor NF-κB via TRAF6 oligomerization (Moscat and Diaz-Meco, 2009). The regulation of inflammatory cytokines secretion by autophagy was mainly demonstrated by measuring the production of active IL-1 and IL-18 by LPS-stimulated ATG16L-deficient mice and macrophages. Knockout of the essential autophagy gene Atg16L1 in mice disrupts basal autophagy and elevates the production of the LPS-induced IL-1β and IL-18 in bone marrow-derived macrophages (BMMs). This reason is that in LPS-stimulated macrophages, depletion of Atg16L1 leads to the activation of caspase-1, which promotes maturation of IL-1 and IL8 by cleaving their precursors (Saitoh et al., 2008). Interestingly, non-invasive Gram-negative bacteria such as *Escherichia coli*, *Enterobacter aerogenes* and *Klebsiella pneumonia*, can also induce IL-1β production by Atg16L1-deficient BMMs, whereas infectious *Salmonella typhimurium* induces produced IL-1β but comparable to non-infectious Gram-negative bacteria, suggesting that the LPS-induced inflammation model is still distinct from true bacterial infection (Saitoh et al., 2008). Similarly, the depletion of Atg16L1 in mouse Paneth cells (a small intestinal epithelial immune cell capable of secreting substances such as intestinal antimicrobial peptides and immunomodulatory proteins) also resulted in a significant increase in LPS-induced transcription of pro-inflammatory cytokines (Cadwell et al., 2008; Lueschow and McElroy, 2020). Harris et al. have demonstrated that stimulation of bone marrow-derived dendritic cells (BMDCs) and immortalized macrophages with LPS resulted in the expression of pro-IL-1β, which was subsequently sequestered into autophagosomes. At this point, if autophagy is further activated with rapamycin, pro-IL-1β is degraded by autophagy and prevents the secretion of other mature cytokines. In contrast, inhibition of autophagy with 3-methyladenine (3-MA) promotes the IL-1β processing and secretion in a NLRP3 (NOD-, LRR- and pyrin domain-containing protein 3)- and TRIF (Toll/IL-1R domain-containing adaptor inducing interferon-β factor)-dependent manner. *In vivo* experiments likewise showed that induction of autophagy with rapamycin reduced serum levels of IL-1β in LPS-stimulated mice. The above data suggest that autophagy controls IL-1β production in at least two ways, directly targeting IL-1β for lysosomal degradation and affecting NLRP3 inflammasome formation (Harris et al., 2011).

The regulation of the inflammation by autophagy through inhibiting the inflammasome has been well recognized (Deretic and Levine, 2018; Seveau et al., 2018; Takahama et al., 2018; Biasizzo and Kopitar-Jerala, 2020). The inflammasome is a cytosolic multi-protein complex consisting of the receptor (mainly NLRs), the adaptor ASC (apoptosis-associated speck-like protein), and the executor caspase-1. Its primary function is to facilitate the processing and shearing of biologically inactive IL-1β and IL-18 precursors into active IL-1β and IL-18 and their secretion, thereby eliciting an inflammatory response. The currently identified inflammasomes include NLRP1 (nod-like receptor protein 1), NLRP3, Ipaf/NLRC4 (NLR containing a caspase recruitment domain 4) and AIM2 inflammasomes, with NLRP3 being the best characterized (Schroder and Tschopp, 2010). Autophagy can both limit the activation of inflammasomes by degrading the inflammasome components and removing damaged organelles caused by a bacterial or viral infection and the ROS they release (Levine et al., 2011; Zhou et al., 2011; Cadwell, 2016; Takahama et al., 2018). It has been found that the autophagy regulatory protein TRIM20 and IRGM (immunity-related GTPase family M) interact directly with components of the inflammasome, hindering their assembly while directing them to the autophagosome for degradation, thereby reducing infection-induced inflammasome activity and inflammatory responses (Kimura et al., 2015; Mehto et al., 2019). Significantly, mutants of IRGM (related to Crohn’s disease) and TRIM20 (related to Mediterranean fever) associated with chronic inflammatory disorders cannot mediate autophagy, suggesting the importance of autophagy for suppressing chronic inflammatory responses and preventing severe inflammatory diseases (Kroemer, 2019). Moreover, mitophagy can reduce damaged mitochondria, thus inhibiting the activation of inflammasome (Yuk et al., 2020). Conversely, autophagy defects lead to intracellular accumulation of damaged mitochondria, increased ROS production, mitochondrial DNA release, and assembly of the NLRP3 inflammasome (Nakahira et al., 2011). Other studies showed the activation of autophagy was dependent on NLRP3 and AIM2 inflammasomes in macrophages (Shi et al., 2012). Notably, many inflammasome agonists, such as silica, alum and monosodium urate crystals, also disrupt lysosomal function, providing additional corroboration that autophagy limits inflammasome activation (Deretic and Levine, 2018).

### THE CROSSTALK OF AUTOPHAGY INFLAMMATION DURING BACTERIAL AND VIRAL INFECTIONS

**FIGURE 2:**
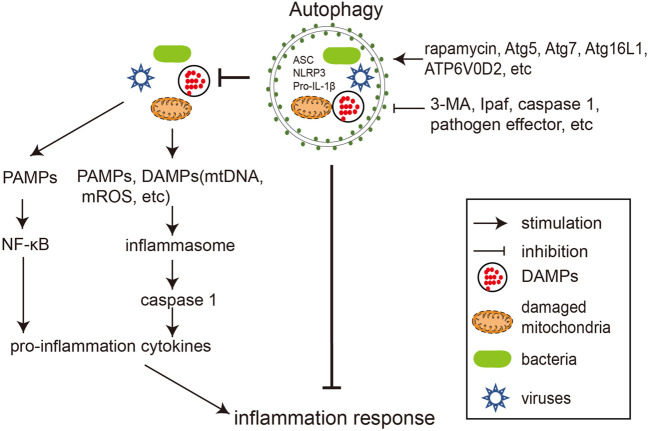
Crosstalks between autophagy and inflammation during pathogen infections. Bacterial and viral infections trigger inflammation by activating NF-κB signaling and inflammasome. Autophagy negatively regulates inflammation by degrading its stimuli, including bacteria, viruses, DAMPs, and inflammasome components. Rapamycin and overexpression of autophagy genes (such as *Atg5*, *Atg7*, *Atg16L1*, and *ATP6V0D2*) reduce inflammation by promoting autophagy, while 3-MA, inflammasome components (e.g., Ipaf, Caspase-1) and pathogen encoded proteins preserve inflammation by antagonizing autophagy.

Autophagy and inflammation are the two arms of the innate anti-infective machinery, and their balanced interaction is essential for maintaining cellular homeostasis and health. Dysfunctional autophagy often leads to uncontrolled inflammatory responses triggered by infection, resulting in severe damage to the host (Deretic, 2021). The crosstalk between autophagy and inflammation during bacterial and viral infections is summarized below in Figure 2.

### The Crosstalk Between Autophagy and Inflammation During Bacterial Infection

Tuberculosis is a globally transmitted chronic infectious disease caused by *Mycobacterium tuberculosis (Mtb)* infection. Macrophages are the primary host cells of *Mtb* and are the main site and the first line of defense against this pathogen. Macrophages can counter *Mtb* invasion through phagocytosis, secretion of various cytokines to initiate inflammation, or through autophagy (targeting free *Mtb* in the cytoplasm). Among these antimicrobial pathways, inflammation and autophagy significantly influence each other. Treatment of cultured mouse macrophages RAW264.7 with the pro-inflammatory cytokine IL-1β resulted in the induction of autophagy and the generation of large numbers of early and mature autophagosomes. Further studies showed that IL-1β treatment reduced *Mtb* in RAW264.7 cells and that the effect of IL-1β on enhancing *Mtb* clearance was attenuated by knocking down the autophagy gene *Atg7*. Treatment of *Mtb*-infected mouse primary BMMs with starvation or IL-1 promoted the clearance of *Mtb* therein, and the amount of *Mtb* cleared was comparable under both treatment conditions. However, both treatments had no effect on *Mtb* clearance in primary BMMs in which the *Atg7* gene was conditionally knocked out. All these data suggest that the inflammatory cytokine IL-1β drives intracellular *Mtb* clearance by initiating autophagy, and subsequent studies have revealed that this signaling pathway is mediated by TBK1 (Pilli et al., 2012). Consistently, conditional knockout of *Atg5* in bone marrow cells (including macrophages and granulocytes) in mice resulted in a significant increase in spleen and lung bacterial load and a shortened survival period upon infection with *Mtb*. At the same time, knockout mice showed signs of inflammation in the lungs, with increased neutrophils and elevated cytokines IL-1α, IL-12, and CXCL1. This study suggests that autophagy can also inhibit *Mtb* growth, excessive inflammation, and the resulting tissue damage in an *in vivo* mouse model (Castillo et al., 2012) (Table 1). However, conflicting conclusions were obtained in subsequent research by Kimmey et al., who suggested that other non-autophagic functions of Atg5 correlate with the outcome of *Mtb* infection. In their study, Atg5 was not required in alveolar macrophages during *Mtb* infection but exerted a unique protective role by preventing PMNs (polymorphonuclear cells)-mediated immunopathology (Kimmey et al., 2015). Recently, researchers have found that treating innate immune cells with the herbal medicine baicalin or vitamin D3 in combination with benzyl butyrate can induce autophagy and control inflammation caused by *Mtb* infection, which was valuable attempt to treat *Mtb* infection pharmacologically (Zhang et al., 2017; Rekha et al., 2018).

**TABLE 1:** The crosstalk between autophagy and inflammation during microbial infection.

Microbial pathogens	Experimental cell and animal model	Inflammation effector	Autophagy-regulator	The function of autophagy-inflammation interplay functions	References
*Mycobacterium tuberculosis*	*Atg7* ^*flox/flox*^ *; LysM-Cre +* mice; murine macrophage cell line, and murine primary bone marrow macrophages (BMMs)	IL-1β, and TBK1	Atg7, Rab8a, and p62	TBK-1 is required for autophagic elimination of mycobacteria in macrophages and IL-1beta activity was dependent on TBK-1	Pilli et al. (2012)
	*Atg5* ^*flox/flox*^ *; LysM-Cre +* mice and murine BMMs	IL-1α, IL-12, and CXCL1	Atg5	Autophagy protects against active tuberculosis by suppressing bacterial burden and inflammation	Castillo et al. (2012)
*Salmonella typhimurium*	*Atg16l1* ^*flox/flox*^ *; Villin-cre* mice and *Atg16l1* ^*flox/flox*^ *; CD11c-cre* mice	IL-1β and IL-6	Atg16L1	Atg16l1 protect mice intestinal epithelial cells from Salmonella infection-related inflammation	Conway et al. (2013)
	*ATP6V0D2* ^*−/−*^ mouse BMMs	IL-1β	ATP6V0D2	ATP6V0D2 restricts inflammasome activation and Salmonella infection by facilitating autophagosome-lysosome fusion	Xia et al. (2019)
*Shigella flexneri*	Hela cell and mouse embryonic fibroblast	NF-κB, IKK, TRAF6, NEMO, CXCL1, and CXCL2	Atg4B, Atg5, and p62	Inflammasome components and caspase-1 are degraded by autophagy in Shigella-infected cells	Dupont et al. (2009)
	Murine BMMs	caspase-1, Ipaf, and IL-1β	ND	Caspase-1 and Ipaf inhibits Shigella-induced autophagy	Suzuki et al. (2007)
*Burkholderia cenocepacia*	Murine BMMs	IL-1β	P62	Autophagy is employed to clear B. cepacian and relieve related inflammation	Abdulrahman et al. (2011); Abdulrahman et al. (2013)
Influenza A virus (IAV)	murine embryonic fibroblast and BMMs	GM-CSF, TNF-α, IL-1β, IL-6, MCP-1, and IL-13	Epg5, Atg14, Fip200, Atg5, and Atg7	Epg5 and other Atg genes function in murine macrophages to limit inflammation in the lung	Lu et al. (2016)
	*RIPK2* ^*−/−*^ mice and murine BMDC	RIPK2, NOD2, NLRP3, IL-18, IFN-γ, and caspase-1	Mitophagy	NOD2-RIPK2 signaling negatively regulates NLRP3 inflammasome activation and IL-18 production via ULK1-dependent mitophagy during IAV infection	Lupfer et al. (2013)
Human immunodeficiency virus 1 (HIV-1)	*GFAP-Tat* transgenic mice	Tat	Rapamycin	Rapamycin-activated autophagy inhibits the neuroinflammation in the Tat-overexpressed mice	Fields et al. (2015)
	Glial cells of mice partially lacking Beclin1	IL-6, RANTES, and MCP-1	Beclin1	Autophagy-deficient (*Beclin1* ^*+/−*^) glial cells had reduced levels of the pro-inflammatory cytokines	Lapierre et al. (2018a)
Murine gammaherpesvirus 68 (MHV68)	*Atg* gene knockout mice	IFN-γ	Fip200, beclin 1, Atg14, Atg16l1, Atg7, Atg3, and Atg5	*Atg* genes impair virus-induced systemic inflammation	Park et al. (2016)
Zika virus	*Drosophila* brain	NF-κB	Atg5, Atg7, and Atg8	Inflammation-induced autophagy restricts Zika virus infection	Liu et al. (2018)
SARS-CoV-2	Human bronchial epithelial and microvascular endothelial cells	TNF-α, IL-6, and IL-8	PI3K/AKT/mTOR	SARS-CoV-2 spike-induced autophagy promotes inflammation in infected cells	Li et al. (2021a)

ND, not determined.

*Salmonella Enterica* is a group of Gram-negative pathogenic bacteria that can cause a variety of diseases in mammals. Among them, *Salmonella Typhimurium* (*S. Typhimurium*) has a broader host range and is an important pathogen of human gastroenteritis. *S. Typhimurium* usually enters the human gastrointestinal tract through the consumption of contaminated food. Upon reaching the small intestine, they break through the mucosal layer into the epithelium and recruit immune cells to the intestinal lumen. The immune cells release cytokines and chemokines and thereby cause local inflammation (gastroenteritis) (Xu et al., 2019). A small fraction of intracellular *S. Typhimurium* becomes the target of autophagy shortly after infection and is thus restricted from growing in the host cell (Zheng et al., 2009). Conway et al. showed that infection of mice with *Salmonella* induced upregulation of the autophagy marker LC3 in gastrointestinal cells and the colocalization of bacterial, Atg16L and LC3. Mice conditionally knockout of Atg16l in the small intestine (*Atg16l1* ^*flox/flox*^ *; Villin-cre*) failed to induce autophagy in their intestinal epithelial cells after infection by *S. Typhimurium*. Compared to wild-type mice, knockout mice were more susceptible to *S. Typhimurium* infection and exhibited more severe cecum inflammation and more bacterial systemic translocation after infection (Conway et al., 2013). Another study using a transgenic mouse model with knockout ATP6V0D2 came to a similar conclusion. ATP6V0D2 is a macrophage-specific isoform of vacuolar GTPase that plays an integral role in autophagosome-lysosome fusion; therefore, its depletion often leads to failed degradation and accumulation of autophagy cargos. Challenging the knockout mice with *Salmonella* results in augmented mitochondrial damage, enhanced inflammasome activation and reduced bacteria clearance in macrophages. In addition, the knockout mice were more susceptible to DSS (dextran sulfate sodium salt) -induced colitis and *Salmonella*-induced death, highlighting the importance of functional autophagy in inflammasome regulation and antimicrobial defense (Xia et al., 2019) (Table 1).

*Shigella flexneri,* the causative agent of human shigellosis, is widely spread in nature, can invade various host cells including epidermal cells, macrophages and dendritic cells. It effectively breaks through the immune defenses, causing hemorrhagic diarrhea and severe intestinal inflammation. During infection, *Shigella* enters host cells through phagocytosis, rapidly escapes from the phagocytic vacuoles mediating the invasion, and grows and multiplies freely in the cytoplasm (Li et al., 2021c). *Shigella* infection has been reported to induce autophagy along with activation of caspase-1 through Ipaf inflammasome, which subsequently promotes the release of inflammatory cytokines IL-1β and IL-18. Knockout of caspase-1 or Ipaf in mouse BMMs leads to increased autophagy during *Shigella* infection, suggesting that autophagy is regulated by the Ipaf inflammasome in macrophages (Suzuki et al., 2007; Suzuki and Núñez, 2008). Meanwhile, a later study by Dupont et al. demonstrated the inflammation is also regulated by autophagy during *Shigella* infection. In *Shigella-*infected epithelial cells, proteins on the phagocytic vacuolar membrane remnants, including the inflammasome components caspase-1 and IKK, were observed to be polyubiquitinated and subsequently recognized and degraded by the autophagosome, which led to an attenuation of inflammation induced by infection. Meanwhile, autophagy dysfunction caused by *Atg4B* mutation or *Atg5* deletion led to the accumulation of p62 and other polyubiquitinated proteins on the phagocytic vacuole membrane and exacerbated early inflammatory and cytokine responses in infected cells (Dupont et al., 2009).

*Burkholderia cepacia* (*B. cepacia*) is an important pathogen of nosocomial infections. It mainly attacks immunocompromised patients, such as those with cystic fibrosis, causing persistent pulmonary inflammation and resistance to almost all antibiotics. Cystic fibrosis is a common and fatal genetic disease in the white population, and approximately 85% of deaths are due to lung infections. Mutations in the *CFTR* (cystic fibrosis transmembrane transduction regulator) gene are the primary cause of cystic fibrosis. In wild-type macrophages, a large number of invading *B. cepacia* are encapsulated by autophagosomes, which subsequently fuse with lysosomes and are degraded. The ΔF508 mutant of pathogenic CFTR can limit autophagosome and lysosome fusion; therefore, *B. cepacia* entering mouse macrophages carrying the CFTRΔF508 mutation are not eliminated by autophagy, leading to an upregulation of IL-1β secretion. Consistently, the *B. cepacia* -infected ΔF508 mice recruit significantly more inflammatory cells to the lung and alveolar spaces than wild-type mice. Rapamycin treatment successfully reversed the morphology observed in *B. cepacia* -infected CFTR ΔF508 macrophages, as evidenced by autophagy restoration, intracellular *B. cepacia* clearance, and reduction of IL-1β secretion. Rapamycin also showed consistent therapeutic effects in *B. cepacia* -infected CFTR ΔF508 mice by inducing autophagy and reducing inflammation and *B. cepacia* load in the lung. Therefore, the above *in vitro* and *in vivo* experiments suggest that promoting autophagy has great potential in treating *B. cepacia* infection in cystic fibrosis patients (Abdulrahman et al., 2011).

### The Crosstalk Between Autophagy and Inflammation During Viral Infection

Influenza A virus (IAV) is a highly infectious, negative-stranded RNA virus that infects both humans and animals. Pneumonia caused by IAV infection is a double-edged sword and requires delicate modulation. When well-controlled, moderate inflammatory cytokines can inhibit viral replication; otherwise, excessive cytokines can lead to lung injury. *Epg5*, an autophagy gene involved in autophagosome/lysosome fusion, was initially identified in *Caenorhabditis elegans* (*C. elegans*) but highly conserved across species from worms to humans. Clinically, recessive mutations in the *EPG5* gene are causally associated with Vici syndrome, a multisystem disorder with abnormal autophagy and varying degrees of impairment of the immune system and recurrent bronchial infections (Cullup et al., 2013; Lu et al., 2016; Wang et al., 2016). An *Epg5*-deficient mouse model that partially recaptured the features of Vici syndrome exhibited elevated baseline innate immune cellular and cytokine-based lung inflammation and was resistant to lethal influenza virus infection. Knockout of other autophagy genes, including *Atg14*, *Fip200*, *Atg5*, and *Atg7* in myeloid cells, also resulted in similar increments of basal pulmonary inflammation and IAV resistance. Thus the susceptibility to IAV can be partly explained by the anti-inflammatory effect of the pulmonary autophagy genes (Lu et al., 2016) (Table 1). A companion paper published by the same group also reported that the same set of *ATG* genes facilitate the reactivation of murine gammaherpesvirus 68 (MHV68) from macrophages by suppressing the excessive systemic inflammation during chronic viral infection (Park et al., 2016). Together, these studies suggest that *ATG* genes share a common function of preventing virus-induced inflammation in the myeloid cells, which can significantly affect infectious diseases. The serine-threonine kinase RIPK2, a key molecule mediating inflammation, also regulates mitophagy by phosphorylating the upstream ULK1 kinase. Compared to wild-type mice, *RIPK2* knockout mice infected with IAV displayed greater susceptibility, impaired mitophagy, increased accumulation of damaged mitochondria and ROS in BMDC cells, more pro-inflammatory cytokines IL-18 and IFN-γ secretion, and a shorter survival period. Glyburide, a specific inhibitor of NLRP3, inhibits IAV-induced caspase-1 activation and IL-18 secretion in BMDC, completely reversing the phenotypic changes brought about by RIPK2 deletion, indicating that the NPLR3 inflammasome is involved in the regulation of inflammation by the RIPK2/mitophagy cascade (Lupfer et al., 2013).

HIV-1 (human immunodeficiency virus-1) is a retrovirus that attacks and gradually destroys the human immune system, leaving the host unprotected in case of infection and leading to acquired immunodeficiency syndromes (AIDs) if left untreated (Kazer et al., 2020). It targets explicitly immune cells such as CD4-positive T cells, macrophages and dendritic cells for replication, generating a widespread inflammation that leads to the death of large numbers of CD4-positive T cells and the release of inflammatory cytokines such as IL-1β, IL-6 and TNFα (Dinkins et al., 2015). In addition, HIV can also cause neuron damage and lead to HIV-associated neurocognitive disorder (HAND), a collective spectrum of neurocognitive diseases in which HIV penetrates the blood-brain barrier and causes neurological damage. HIV-1 TAT, a regulatory protein essential for the transactivation of HIV dsDNA transcription, is usually secreted by infected cells of the central nervous system (CNS) and transported from the periphery back to the brain through the blood-brain barrier, is toxic to various CNS cells, and is widely recognized as a potential factor in HAND (Ruiz et al., 2019; Marino et al., 2020). Neuroinflammation increased in transgenic mice overexpressing Tat protein. When the mice were treated with the autophagy activator rapamycin, neuroinflammation decreased, indicating that autophagy suppressed neuroinflammation induced by overexpression of Tat protein (Fields et al., 2015). Autophagy-defective (*Beclin1* ^*+/−*^) glial cells exposed by Tat protein down-regulates the inflammatory cytokines released by the cells, which suggests that Beclin1 or autophagy may promote the release of inflammatory cytokines induced by Tat protein (Lapierre et al., 2018b). These two studies provided the opposite example of regulation between inflammation and HIV Tat protein-regulated autophagy. In-depth exploration in this direction is expected to clarify the cause of neuroinflammation in HAND.

Herpes simplex viruses (HSVs) are enveloped double-stranded DNA viruses that primarily invade tissues of ectodermal origin, including skin, mucous membranes, and nerves. The diversity of sites of infection and diseases caused and the tendency to establish latent infections make HSV a severe threat to human health. In a chronically HSV infection, latent viruses are constantly reactivated to produce infectious viral particles (Cohen, 2020). Conditional deletion of multiple *Atg* genes in the myeloid compartment resulted in greater systemic immune responses, increased IFN-γ secretion in T cells and IFN-γ induced transcription in macrophages, and defective viral reactivation from latency in mice chronically infected with one kind of HSV -MHV68. Neutralization of IFN-γ partially rescued the defect in latent virus reactivation in the knockout mice. Interestingly, the knockout of *Atg* genes did not affect viral replication and the establishment of latency. Thus, this study suggests that autophagy genes promote MHV68 reactivation by suppressing virus-induced systemic inflammation rather than triggering intrinsic responses within infected cells. However, whether it is the canonical autophagic function or other non-autophagic functions of the *Atg* genes involved in this process requires further investigation (Park et al., 2016).

Zika virus belongs to the flavivirus and is associated with multiple neurological symptoms (Ledur et al., 2020). Liu et al. reported that the adult Drosophila brain infected with the Zika virus protects itself by activating NF-κB inflammatory signaling to overexpress the dSTING protein and induce autophagy. Blocking autophagy leads to more severe Zika infection in the brain and increased mortality in Drosophila, while pharmacological activation of autophagy is protective from the damage (Liu et al., 2018) (Table 1). However, similar experiments to elucidate the interaction of autophagy and inflammation during Zika virus infection in mammals are yet to be performed.

The recent SARS-CoV-2 infection caused a devastating pandemic of COVID-19, claiming more than five million lives already worldwide. A burst of pro-inflammatory cytokines such as IL-6, TNF-α, G-CSF, IL-1β, and IL-7 secretion caused by the SARS-CoV-2 invasion and excessive inflammation in the lungs, often referred to as a cytokine storm, is thought to be a major cause of death in patients with COVID-19 (Hunter, 2020). It has been reported that SARS-CoV-2 spike protein can induce autophagy in human bronchial epithelial cells and microvascular endothelial cells by elevating intracellular ROS, thereby inhibiting PI3K/AKT/mTOR. The overactivated autophagy then promotes inflammation and apoptosis in infected cells, and the exact mechanism remains to be explored (Li et al., 2021a). Another study showed that SARS-CoV-2 could use the ORF3a protein to weaken STX17-SNAP29-VAMP8 SNARE, a complex essential for autophagosome maturation, to inhibit autophagy and increase replication (Miao et al., 2021). The autophagy inhibitors chloroquine (CQ) and hydroxychloroquine (HCQ) can also inhibit SARS-CoV-2 replication in cultured African green monkey kidney Vero cell line (Liu et al., 2020; Wang et al., 2020). The antiviral effects of CQ and HCQ in Vero cells were not reproduced in human respiratory epithelial cells and macaques (Hoffmann et al., 2020; Maisonnasse et al., 2020). Moreover, clinical trials on anti-SARS-CoV-2 with CQ/HCQ were discontinued because the acceptable dose of CQ/HCQ for patients had no antiviral effect and had toxic side effects (Boulware et al., 2020; Ferner and Aronson, 2020; Ghazy et al., 2020). HQ/HCQ are not specialized autophagy inhibitors. They inhibit autophagy and limit viral invasion by the exact mechanism that neutralizes the low pH environment of endosomes and lysosomes. Therefore the therapeutic result of HQ/HCQ on COVID-19 is also not equivalent to the effect of inhibiting autophagy. Therefore, further research is needed to manipulate autophagy to combat viral infections, and progress in this area is highly dependent on the development of new specific autophagy inhibitors.

## Summary and Outlook

During bacterial and viral infections, autophagy has an important role in regulating inflammation, which in turn regulates autophagy. Although some manifestations and pathways of the crosstalk between autophagy and inflammation have been preliminarily elucidated, the mechanisms of their mutual regulation are not yet fully understood. Recent advances in proteomics and genome-wide gene disruption technologies have facilitated large-scale screening and analysis of the interactions between autophagic core proteins and inflammatory molecules, accelerating the understanding of autophagy/inflammation crosstalk.

Knowledge of the interplay between pathogen-stimulated inflammation and autophagy at the molecular level will facilitate therapeutic interventions against infectious diseases. Many factors are involved in the autophagy/inflammation battlefield between pathogens and hosts, and undoubtedly many more remain to be discovered. Autophagy is known to regulate inflammation by removing pathogens and broken organelles that act as inflammatory triggers, by directly removing inflammasome components, and by regulating organelle function within immune cells to influence inflammation indirectly; accordingly, inflammatory cytokines and inflammasome components such as SPMs can also target various steps of the autophagic machinery, inhibiting or hijacking autophagy to promote inflammatory responses. A more refined understanding of the spatial and temporal processes and factors involved in the inflammatory/autophagic cascade during pathogen invasion will guide the selection of appropriate timing and means of intervention to manipulate autophagy, control inflammation, and prevent infection. Currently, studies on content-dependent autophagic/inflammatory crosstalks during infection with specific pathogens, especially highly pathogenic viruses such as HIV, Zika virus and SARS-CoV-2, are generally lacking and require extra attention.
